# Can NMDA Spikes Dictate Computations of Local Networks and Behavior?

**DOI:** 10.3389/fnmol.2019.00238

**Published:** 2019-09-27

**Authors:** Elisabete Augusto, Frédéric Gambino

**Affiliations:** UMR5297 CNRS Centre Broca Nouvelle-Aquitaine, Interdisciplinary Institute for NeuroScience, University of Bordeaux, Bordeaux, France

**Keywords:** dendritic integration, neuronal network, synaptic plasticity, behavior and cognition, dendritic spikes

## Abstract

Intelligence is the ability to learn appropriate responses to stimuli and the capacity to master new skills. Synaptic integration at the dendritic level is thought to be essential for this ability through linear and non-linear processing, by allowing neurons to be tuned to relevant information and to maximize adaptive behavior. Showing that dendrites are able to generate local computations that influence how animals perceive the world, form a new memory or learn a new skill was a break-through in neuroscience, since in the past they were seen as passive elements of the neurons, just funneling information to the soma. Here, we provide an overview of the role of dendritic integration in improving the neuronal network and behavioral performance. We focus on how NMDA spikes are generated and their role in neuronal computation for optimal behavioral output based on recent *in vivo* studies on rodents.

The appropriate integration of various inputs is fundamental for perceiving the world and for adequate learning. Most of the excitatory synaptic inputs in pyramidal neurons are located in the dendrites, namely on thin dendrites where the majority of the spines are located (Larkman, 1991). Thus, thin dendrites play a crucial role in synaptic integration and plasticity (reviewed in Major et al., 2013), since they are able to exhibit local membrane potential dynamics (Schiller et al., 1997; Schiller et al., 2000) and transform the spatio-temporal sequences of inputs into an output pattern (Polsky et al., 2004; Larkum et al., 2009; Branco et al., 2010). Therefore, understanding the transformation of synaptic inputs to output [e.g., action potentials (APs), “plateau potentials”] requires a deep understanding of the intrinsic physiological properties of dendrites, namely the dendritic compartmentalization, signal transformation and regenerative properties that shape how the spatio-temporal combination of inputs are computed (Major et al., 2013). This review explores what the *in vivo* studies tell us about the impact of the generation of NMDA spikes on pyramidal neurons in animal’s behavior.

## Regenerative Properties of Thin Dendrites – NMDA Spikes

**FIGURE 1:**
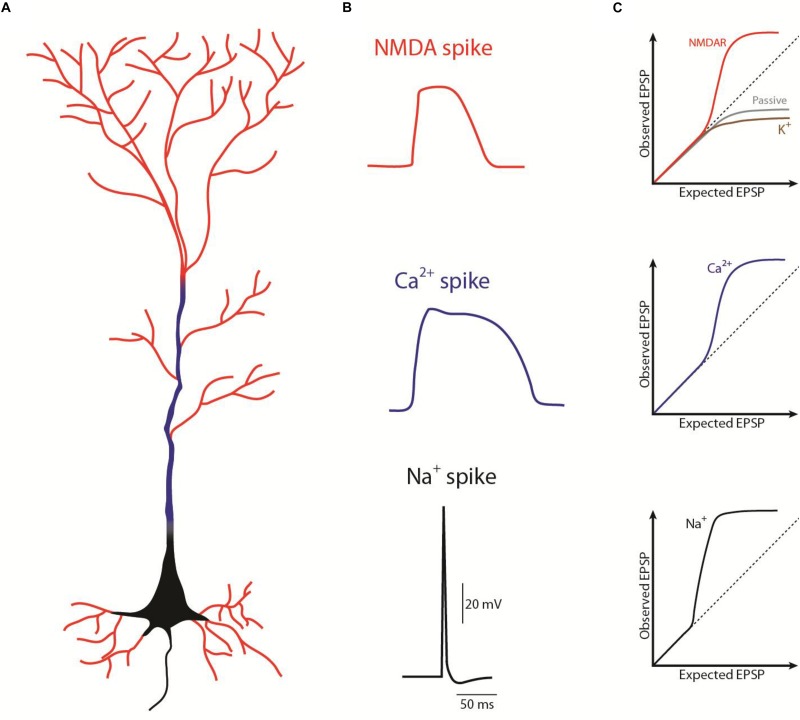
Schematic representation of the different spikes in a pyramidal neuron. **(A)** Representation of where the different spikes take place: NMDA spikes (red) in “thin” dendrites, Ca^2+^ spikes (blue) in “thick” dendrites, Na^+^ spikes or action potential (black) in the axon (as in Larkum et al., 2009). **(B)** Waveform of the different spike (as in Antic et al., 2010). **(C)** Contribution of the different ion channels on the excitatory postsynaptic potentials (EPSP) summation; supralinear summation above the dashed line, sublinear summation bellow the dashed line. EPSP summation properties depends not only on the ion channels but on the combination between those, morphology (dendritic diameter, distance from soma, and branchpoints) and synaptic strength. A combination of those can give rise, for example to a linear summation (as in Tran-Van-Minh et al., 2015).

The pyramidal neurons receive the majority of excitatory glutamatergic synaptic inputs through dendritic spines (Lüscher et al., 2000), which contain various ions-permeable channels. Among the wide range of ionotropic glutamate receptors, AMPA receptors in the spine mediate depolarization with fast decay, but that may promote the release of Mg^2+^ that blocks NMDARs (Hao and Oertner, 2012). NMDA receptors (NMDARs) are also glutamate receptors abundant in the dendritic spines (Sabatini et al., 2002) and are highly permeable to Ca^2+^ and Na^+^ (Sabatini et al., 2002), mediating the majority of the postsynaptic Ca^2+^ influx during synaptic depolarization (Koester and Sakmann, 1998; Schiller et al., 1998). NMDAR activation mediates a slow current that persists for tens to hundreds of milliseconds (Popescu et al., 2004). As a result, synaptic inputs can, in certain conditions, trigger regenerative dendritic events that may be long-lasting (reviewed in Antic et al., 2010 and Major et al., 2013), therefore also termed “plateau potentials” or NMDA spikes (Figure 1).Moreover, the dendritic shaft holds voltage-gated Ca^2+^ channels (Magee and Johnston, 1995) and extrasynaptic NMDARs that may play a role in glutamate spillover during high-frequency activation and may amplify and spread the synaptically mediated depolarization toward the dendritic branch(Chalifoux and Carter, 2011). Distal individual synapses have a weak impact on the initial segment of the axon, since distal synaptic events undergo considerable voltage attenuation as they propagate along the dendrite (Nevian et al., 2007; Larkum et al., 2009). However, this filtering phenomenon is influenced by eventual correlations in time and space between the synaptic events. For instance, when a dendritic branch receives sparse synaptic inputs correlated in time, the information seems to be integrated in a linear mode (Figure 1C; Mel, 1993), in which there is little cooperativity between simultaneously activated synaptic inputs. However, when there is a pronounced spatio-temporal cooperativity between the synaptic inputs (i.e., a high correlation between the timing and the location on the dendritic branch of the onset of the synaptic inputs), these can trigger a non-linear or supralinear summation, that generates a depolarization of the dendritic branch (Figure 1; Llinás et al., 1968; Mel, 1993; Schiller et al., 1997; Schiller et al., 2000; Losonczy and Magee, 2006; Larkum et al., 2009). As a result, during strong glutamatergic release when glutamate binds to NMDARs and the Mg^2+^ block site is released, the NMDAR current can potentially fire a regenerative NMDA spike on the dendrite (see review Antic et al., 2010).

The ionic mechanism of a NMDA spike is very dynamic since their properties (i.e., their threshold, duration in time and length on the dendritic branch) depends on the baseline membrane potential (Polsky et al., 2009). For instance, the number of activated spines triggering a NMDA spike should change as a function of baseline membrane potential, because depolarization reduces the NMDA spike threshold (Major et al., 2008), by lowering the required glutamate to bind to NMDAR. This means that the generation of a NMDA spike depends on the depolarization drive of the dendrite, which can be provided not only from the glutamate from previous activation, but also from the cooperativity between the different dendritic integration phenomenon, like: (i) a previous NMDA spike (Polsky et al., 2009), (ii) a NMDA spike at a more distal location in the same dendrite (Branco et al., 2010; Behabadi et al., 2012), (iii) a distributed NMDA spike that spread out over the group of dendritic branches (Lavzin et al., 2012), or (iv) a back-propagating-action potential that invaded that branch (Stuart et al., 1997). Additionally, the duration of the NMDA spikes increases linearly with the intensity of the glutamatergic stimulation (Milojkovic et al., 2005a, b). This is a way to compute the intensity of the stimulus that is not amplitude-dependent. It is an important parameter, since it potentially increases the time window to integrate and link fragmented information, such as those arising from different sensory modalities or arriving at the different dendritic regions of the neuron, a phenomenon known as temporal binding and further explored in this review. Additionally, because NMDA spikes are ligand dependent, i.e., dependent on glutamate and D-serine or glycine, they depend not only on local membrane potential but also on the timing and the spatial distribution of these transmitters along the dendrite (reviewed in Major et al., 2013). As a result, the NMDA spikes may act as a detector of synchronous pre- and post-synaptic activity (Waters et al., 2003).

The description of NMDA spikes represented an important break-through in the field, opening the window onto the dramatic impact of distal synaptic inputs on the neuron output. In fact, the different local processing and computations that occur at the dendritic level determine how electric signals propagate and their interaction between different dendritic regions. For example, NMDA spikes can either be restricted to a branch, by failure of active propagation at the branchpoint (Golding et al., 2002; Remy and Spruston, 2007), or they can spread regeneratively to the soma to influence axonal output (Larkum et al., 2009). The cooperative and active integration properties in the dendrites (Figure 1C) further support the idea that NMDA spikes depend on recent and ongoing activity in the local network and may serve as a powerful mechanism to modify the network by inducing the long-term strengthening of co-activated neighboring inputs (Schiller et al., 2000; Cichon and Gan, 2015). In agreement, it has been suggested that neurons capable of firing NMDA spikes can exhibit a greater specificity of spiking responses and perform a greater number of transformations of synaptic input into an AP output, which would otherwise require more than one neuron with passive dendrites (Larkum and Nevian, 2008). In conclusion, NMDA spikes are the putative substrate for the multiple and simultaneous computations at different sites that one pyramidal neuron can perform, thereby increasing the computational power and the repertoire of these cells.

## The Drive of NMDA spikes *In Vivo* – Input Clustering?

Pyramidal neurons have complex dendritic arborizations that receive different inputs targeting spatially separate regions of the neuron. For instance, a cortical network relies on different layers of processing arriving to the different regions of the neuron, from local intracortical, long-range corticocortical and subcortical projections, with the putative influence of inhibition and neuromodulation at each of these connections (Roelfsema and Holtmaat, 2018). But what do we know about what drives a NMDA spike *in vivo*? Early *in vitro* and *in silico* work proposed that inputs with similar information content are spatially clustered in the dendrites (Iannella and Tanaka, 2006; Losonczy et al., 2008), and that active synaptic inputs clustered within a group of spines close to each other on the same dendritic segment were required to generate a NMDA spike (Polsky et al., 2004; Larkum et al., 2009). Additional *in vitro* and *in silico* studies suggested that NMDA spikes in distal dendrites can be evoked by as few as ∼10 clustered spines or by 20 inputs distributed sparsely along a longer dendrite (Major et al., 2008), supporting the idea that clustering is not a prerequisite to trigger a NMDA spike.

Anatomical studies *in vivo* support the idea that inputs onto dendrites are not random, and can be clustered onto specific dendritic branches in pyramidal neurons of the hippocampus (Druckmann et al., 2014), on layer 2/3 of somatosensorial cortex (Makino and Malinow, 2011), and on layer 5 of the motor cortex (Fu et al., 2012). In agreement, *in vivo* functional studies from layer 2/3 pyramidal neurons in the somatosensorial cortex, support the idea that spontaneous synaptic inputs are often synchronized reaching a group of spines in the vicinity of each other (Takahashi et al., 2012), and that clustered plasticity may also result from interspine interactions (Harvey and Svoboda, 2007), since local depolarization-induced Mg^2+^ unblock of nearby NMDARs decreases the threshold for a regenerative membrane potential event (Losonczy et al., 2008). In agreement, it was proposed that functionally similar synaptic inputs clustered in space and time into dendrites of layer 2/3 neurons of the visual cortex, correlates with dendritic events and predicts orientation selectivity *in vivo* (Wilson et al., 2016) On the other hand, the work of Lavzin et al. (2012) suggests that *in vivo* sparse stimulation of two different inputs (pairing of corticocortical and thalamocortical inputs) or with focal glutamate uncaging in spiny stellate neurons from layer 4 of the somatosensorial cortex can generate NMDA spikes. In agreement with the non-clustered hypothesis, the work by Arthur Konnerth’s lab indicates that single-spine responses evoked by similar sensory information *in vivo*, are dispersed across multiple dendritic branches of layer 2/3 neurons of the visual cortex (Jia et al., 2010), somatosensorial cortex (Varga et al., 2011), and auditory cortex (Chen et al., 2011). In conclusion, *in vivo* studies provide evidence that dendritic inputs are not random and can be dispersed or clustered (Iacaruso et al., 2017), possibly depending on the local network and stimulation modality. Importantly, the resultant input organization with presynaptic synchrony or spines in the vicinity of each other integrating different information may offer opportunities to encode complex associative learning processes at the dendritic level.

## Dendritic Spikes and *In Vivo* Synaptic Plasticity

After the description of NMDA spikes, important work unraveled the role of these events in synaptic plasticity. As mentioned above, most pyramidal neurons receive at least two functionally distinct inputs – long-range afferents mainly contacting the distal apical dendrites, and local inputs innervating proximal perisomatic dendrites. The active dendritic mechanisms, such as NMDA spikes, allow the integration and potential interaction of the various afferents, if streaming with appropriate time-coincidence, thereby opening the window for important events in synaptic plasticity. Studies showing the importance of other types of dendritic events such as back-propagating AP on synaptic plasticity, namely spike-timing-dependent plasticity (Sjöström and Häusser, 2006), are very relevant but are beyond the scope of this review.

*In vitro* studies have shown that the CA1 pyramidal neurons of the hippocampus can generate NMDA spikes through the integration of CA3 inputs and entorhinal cortex afferent (Remy and Spruston, 2007; Takahashi and Magee, 2009). These regenerative events are thought to trigger synaptic potentiation through the influx of calcium into the post-synaptic compartment without requiring an AP (Golding et al., 2002). These studies show that the initiation of NMDA spikes can induce rapid and long-lasting changes in synaptic strength and change the intrinsic excitability of dendrites. Importantly, the combination of various afferents by the dendrites points to the generation of neurons that putatively have functional feature selectivity to both inputs, generating networks with higher computational power. To further understand this phenomenon, researchers have explored whether they were present *in vivo*. Gambino et al. (2014) showed that whisker deflection triggers NMDAR-mediated long-lasting depolarizations. This was dependent on the integration of different inputs, namely thalamocortical inputs into the tuft dendrites of layer 2/3 pyramidal neurons of the barrel cortex, producing “plateau potentials” in the absence of somatic spiking (Gambino et al., 2014). The “plateau potential” is an important event for the induction of synaptic plasticity, a mechanism that may prevent cortical neurons from losing synaptic inputs. Gambino et al. (2014) were the first to demonstrate long-term potentiation *in vivo* that does not require AP, but is instead dendritic and NMDA-dependent. A similar phenomenon was described in CA1 pyramidal neurons of the hippocampus, in which integration of inputs from the entorhinal cortex and CA3 at the dendritic level was able to trigger a ramp of membrane potential depolarization associated with a position-specific increase of synaptic weight and sufficient to induce a place field formation (Bittner et al., 2015, 2017). Additionally, these studies indicate that input-potentiating plasticity and not increase in input numbers are determinant for that phenomenon (Bittner et al., 2015). Altogether, these studies show that dendritic computation of different afferents is able to trigger a single “plateau potential” that is sufficient to increase the synaptic weight of the excitatory inputs, thereby allowing the maintenance of essential spines (Gambino et al., 2014) or the abrupt formation of new CA1 place fields (Bittner et al., 2015, 2017). These are crucial events for correctly perceiving the environment and having adequate memory storage.

Finally, it is important to realize that neuromodulators and inhibition can have a direct effect on the active properties of dendrites and that the intrinsic properties of dendrites are also subject to plasticity (Frick and Johnston, 2005; Roelfsema and Holtmaat, 2018). Hence, those mechanisms provide additional ways by which synaptic plasticity can influence the effect of synaptic input on neuronal output. Nevertheless, the *in vivo* studies seem to be in agreement with what has been shown *in vitro* and *in silico*, showing that individual dendritic branches serve as a basic unit for synaptic plasticity and possibly involved in information storage.

## Impact of Dendritic Spikes on Behavior

Since the demonstration of the role of NMDA spikes on input integration, amplification and computation in the cortex and hippocampus the impact of these events on behavior performance has become a central focus of research. The relationship between dendritic activity and sensorial perception began to be probed at the beginning of the present decade. One of the first studies using dendritic Ca^2+^ imaging in awake mice showed that sensorial stimulation of the hindlimb could drive regenerative dendritic events in the apical tuft of layer 5 neurons in the hindlimb somatosensorial cortex (Murayama and Larkum, 2009). Using whole-cell recordings in anesthetized mice, Lavzin et al. (2012) showed that dendrites of layer 4 spiny stellate neurons in the barrel cortex integrate different inputs (thalamocortical and corticocortical) supralinearly, generating NMDA spikes that reflect angular whisker tuning. Since different anesthetics can induce the shutdown of important inputs that potentially drive dendritic activity, both anesthetized and awake animals were compared by using whole-cell patch clamp and imaging recordings *in vivo*, showing that both conditions exhibited dendritic events with similar trends. For example, Smith et al. (2013) showed that in both conditions (lightly anesthetized and awake) visual inputs trigger NMDA spikes in the tuft dendrites of layer 2/3 neurons in the visual cortex, a mechanism that tuned those neurons to specific orientation. NMDA spikes were also observed in the hindlimb somatosensorial cortex triggered by electrical stimulation of the contralateral hindpaw (Palmer et al., 2014). Recently, significant studies reported dendritic plateaus during active behavior rodents. For example, Xu et al. (2012) reported dendritic nonlinearity events in the apical tuft dendrites of layer 5 pyramidal neurons of the barrel cortex during an active sensing behavior that required the integration of sensory and motor information.

Owing to the role of the CA1 region of the hippocampus in place field generation and spatial memory, *in vivo* work was performed on its pyramidal neurons. By combining whole-cell recordings and dendritic Ca^2+^ imaging, it was shown that NMDA spikes of CA1 hippocampal pyramidal neurons were required to trigger high-frequency bursting *in vivo* (Grienberger et al., 2014). Bittner et al. (2015), not only corroborated the finding that the “plateau potentials” were sufficient to induce place field formation in CA1 pyramidal cells *in vivo*, but they also confirmed their role in the strengthening of synaptic inputs and that they were driven by the integration of specific long-range inputs.

The elegant work of Cichon and Gan (2015) raised much excitement in the field by showing branch specificity on NMDA spikes, the impact of the latter on spine dynamics and, very importantly, the causality between these events and behavioral performance. Briefly, they showed that different running tasks induced NMDA spikes on different branches of the tuft dendrites of the same neurons of the motor cortex (controlled by cortical inhibition), and that these branch-specific spikes led to a long-lasting increase in the strength of synapses that were active at the moment of NMDA spike generation (Cichon and Gan, 2015). Previous *in vitro* studies have shown that NMDA spikes can cause either synaptic potentiation or depotentiation, depending on the time interval between synaptic activity and spike generation (Lisman and Spruston, 2005; Sjöström and Häusser, 2006). However, by showing the spatial segregation of NMDA spikes on different tasks, this study showed how synaptic changes induced by new experiences reduce the chance of disrupting what was acquired in past experiences. In summary, the authors demonstrated the importance of branch-specific NMDA spikes in maintaining experience-dependent synaptic plasticity, and consequently its role in learning.

Another important study showed the causality between dendritic integration and behavior performance (Takahashi et al., 2016). The authors showed that the inhibition of dendritic events in the somatosensorial cortex was sufficient to decrease sensorial perception in mice (Takahashi et al., 2016). It also showed that the threshold for sensorial perception depends on dendritic mechanisms with the participation of somatostatin interneurons (Takahashi et al., 2016).

In conclusion, *in vivo* studies to date have provided evidence of the ability of the dendrites of pyramidal neurons to actively integrate inputs from spatially segregated and functionally distinct pathways whenever strong temporal correlations exist between these representations. The triggered regenerative events can amplify the effects of inputs that correlate with the detection of stimuli (Takahashi et al., 2016), a memory (Bittner et al., 2015) or a skill that was learned (Cichon and Gan, 2015), contributing to a higher cognitive performance.

## Conclusion and Open Questions

We now know that dendrites have the capacity to influence how neurons integrate their inputs. Depending on the morphology, the passive and active properties of the dendrites, the synaptic strength and the specificity of their inputs, dendrites are capable of integrating information with passive interaction (resulting in sublinear summation along the dendritic tree); or with active integration, by processing the inputs nonlinearly and generating regenerative spike-like events (Figure 1C; Tran-Van-Minh et al., 2015). Thus, dendrites are capable of a wide range of computations and dendritic interactions increasing the array of transformations of synaptic inputs into output (“plateau potencial,” action potential or burst).

The different *in vivo* studies corroborate the ability of dendrites of pyramidal neurons to actively integrate inputs from spatially and functionally distinct pathways when temporal correlations exist between them. The triggered regenerative events (i.e., NMDA spikes) may serve as a powerful mechanism to modify the network by inducing long-term strengthening of co-activated inputs (Gambino et al., 2014; Cichon and Gan, 2015; Bittner et al., 2017). In agreement, it was shown that dendrites are required to amplify the diverse inputs that correlate to sensorial perception (Takahashi et al., 2016), a memory (Bittner et al., 2015) or a new skill that was learned (Cichon and Gan, 2015). Thus, the capacity to integrate different information may offer opportunities to encode complex associative learning processes at the dendritic level. This hypothesis is in line with the idea that circuit computations based on active dendritic transformations of different streams of information are the potential substrate for the multiple and simultaneous computations at different sites that one pyramidal neuron can perform. This underlies the variety of functions necessary in high cognitive performance, including top-down cortical interactions, associative feature binding and predictive coding.

Urge by technological advances, future *in vivo* research will increase our knowledge on the intricate role of dendrites on brain’s computations. Further *in vivo* studies exploring the impact of inhibition and neuromodulation, as well as the anatomical organization and functional spatio-temporal interaction of the different inputs, on dendritic computation and local network would allow us to better understand the generation and the impact of these events in behaving subjects. In particular, further research exploring under which conditions neurons generate dendritic spikes *in vivo*, i.e., how activity in multiple presynaptic pathways (and not only two) is integrated during a variety of behaviors, namely in high cognitive demand tasks, by (i) exploring the convergence of multiple synaptic inputs carrying different information (Petreanu et al., 2012; Lovett-Barron et al., 2014) and of dendritic and somatic activity simultaneously; (ii) scanning the role of inhibition and neuromodulation on these events; and (iii) study the cooperativity between spines or dendritic branches of the same neuron. These studies can eventually be propelled by the recent development of different probes for *in vivo* imaging, namely different calcium indicators (Fosque et al., 2015; Dana et al., 2016), glutamate (Marvin et al., 2018), dopamine (Patriarchi et al., 2018), and voltage-sensitive sensors (Adam et al., 2019), together with the fast advances on the imaging field, allowing faster and deeper volume imaging (Vogt, 2016; Wang et al., 2018). Another important question in the field is to understand how the different types of dendritic integration relate to brain function. This can eventually be answered, not only by exploring how behavior correlates with dendritic activity and modulates their intrinsic properties, but with a tool that would allow researcher to very precisely (spatio-temporaly) control the dendritic activity of specific segments of the dendrite during behavior (Carmi et al., 2019). A similar mechanism may be possible to test in humans in the future, since it was shown that transcranial magnetic stimulation can noninvasively suppress Ca^2+^ activity in pyramidal dendrites (Murphy et al., 2016).

## Author Contributions

EA and FG equally contributed to this work.

## Conflict of Interest

The authors declare that the research was conducted in the absence of any commercial or financial relationships that could be construed as a potential conflict of interest.
